# Single cell analysis unveils B cell-dominated immune subtypes in HNSCC for enhanced prognostic and therapeutic stratification

**DOI:** 10.1038/s41368-024-00292-1

**Published:** 2024-04-16

**Authors:** Kang Li, Caihua Zhang, Ruoxing Zhou, Maosheng Cheng, Rongsong Ling, Gan Xiong, Jieyi Ma, Yan Zhu, Shuang Chen, Jie Chen, Demeng Chen, Liang Peng

**Affiliations:** 1 State Key Laboratory of Oncology in South China, Department of Oral and Maxillofacial Surgery; Institute of Precision Medicine; Center for Translational Medicine, The First Affiliated Hospital of Sun Yat-sen University, Guangzhou, China; 2 Institute for Advanced Study, Shenzhen University, Shenzhen, China; 3 Hospital of Stomatology, Guangdong Provincial Key Laboratory of Stomatology, Guanghua School of Stomatology, Sun Yat-Sen University, Guangzhou, China; 4 Senior Department of Oncology, The Fifth Medical Center of PLA General Hospital, Beijing, China

**Keywords:** Cancer models, Cancer microenvironment

## Abstract

Head and neck squamous cell carcinoma (HNSCC) is characterized by high recurrence or distant metastases rate and the prognosis is challenging. There is mounting evidence that tumor-infiltrating B cells (TIL-Bs) have a crucial, synergistic role in tumor control. However, little is known about the role TIL-Bs play in immune microenvironment and the way TIL-Bs affect the outcome of immune checkpoint blockade. Using single-cell RNA sequencing (scRNA-seq) data from the Gene Expression Omnibus (GEO) database, the study identified distinct gene expression patterns in TIL-Bs. HNSCC samples were categorized into TIL-Bs inhibition and TIL-Bs activation groups using unsupervised clustering. This classification was further validated with TCGA HNSCC data, correlating with patient prognosis, immune cell infiltration, and response to immunotherapy. We found that the B cells activation group exhibited a better prognosis, higher immune cell infiltration, and distinct immune checkpoint levels, including elevated PD-L1. A prognostic model was also developed and validated, highlighting four genes as potential biomarkers for predicting survival outcomes in HNSCC patients. Overall, this study provides a foundational approach for B cells-based tumor classification in HNSCC, offering insights into targeted treatment and immunotherapy strategies.

## Introduction

Head and neck cancers (HNC) consist of a variety of tumors of the upper respiratory tract and are the seventh most common cancer worldwide.^1,2^ And among HNC, head and neck squamous cell carcinoma (HNSCC) is the most common histologic subtype, accounting for approximately more than 90%.^3^ Over the past decades, multimodal approaches based on surgery, radiotherapy, and molecular targeted therapy have provided substantial clinical benefit to patients with HNC. Unfortunately, a high percentage of patients still end up with recurrence or distant metastases, and their prognosis remains poor.^4–6^ And as a first-line treatment for patients with progressive recurrence or metastasis, immune checkpoint blockade therapy has revolutionized the treatment strategy.^7,8^ Yet in-depth studies are needed regarding how to optimize the therapy with these agents, such as improving the overall response rate of patients. Therefore, HNSCC remains an extremely complex group of diseases and at this stage a better understanding of the molecular and cellular mechanisms of HNSCC is needed, which may help to discover new therapeutic strategies.

The tumor microenvironment (TME) consists of a variety of immune cells, endothelial cells, and fibroblasts, with which tumor cells interact continuously during tumor development.^9^ And there is growing evidence that the ongoing interaction between tumor cells and the tumor microenvironment is an important determinant of tumorigenesis, progression, metastasis, and response to therapy.^10,11^ For example, in melanoma, higher densities of CD8^+^ T cells in the tumor core and margin are associated with increased PD-1 and PD-L1 blockade responses,^12^ while several subpopulations of CD4^+^ Th1 cells are also more abundant in CTLA-4-responsive tumors.^13^ And in recent studies, intratumoral or peritumoral B cells have been associated with a positive prognosis and response to immunotherapy.^14–16^ These B cells usually form tertiary lymphoid structures (TLS) that establish a local and sustained immune response, exerting specific antitumor immune effects by secreting antibodies that recognize tumor-associated antigens and enhancing the killing action of T cells and natural killer cells.^14,16^ In HNSCC, tumor-infiltrating B lymphocytes (TIBs) are an important component of TME, indicating their involvement in the development of HNSCC. Furthermore, it has been shown that TIBs are associated with better patient survival in HNSCC^17^ and that B-cell activation is associated with PD-1 blockade.^18^ These studies suggest that the B-cell population plays a critical role in antitumor immunity in HNSCC, and therefore it is necessary to explore the gene expression profile of B cells in HNSCC and its relationship to patient prognosis and immunotherapy prediction.

Recently, single-cell RNA sequencing (scRNA-seq) technology has provided an efficient way to obtain high-resolution portraits of tumor cell ecosystems from tumors.^19,20^ More specifically, scRNA-seq analysis of cell types and cell states of the TME can provide further information on the molecular characteristics of the disease, paving the way for personalized immunotherapy. Establishing tumor subtype typing based on the molecular characteristics of specific immune cells may be a reliable way to predict the effect of immunotherapy as well as the prognosis of patients. Here, we characterized the scRNA-seq dataset of HNSCC regarding B cells and established two B cell subtypes based on single-cell expression of differential genes. We characterized both subtypes at the single-cell level and at the gene level; and verified the consistency of typing in an additional independent sample cohort. In addition, we analyzed the differential genes of the two B-cell subtypes and constructed a B-cell activation gene signature. We then validated the prognostic value of the B-cell activation gene signature based on the TCGA database and the GEO database, and analyzed in depth its relationship with immune infiltration and ICB response. In addition, we pioneered the application of HNSCC in combination with Cox proportional hazards model to establish a prognostic risk prediction model. This study provides support for the role of B cells in antitumor immunity in HNSCC and provides new insights into B cell-based tumor classification.

## Results

### Identification and characterization of B-cell immune classification in an independent scRNA-seq dataset

**Fig. 1:**
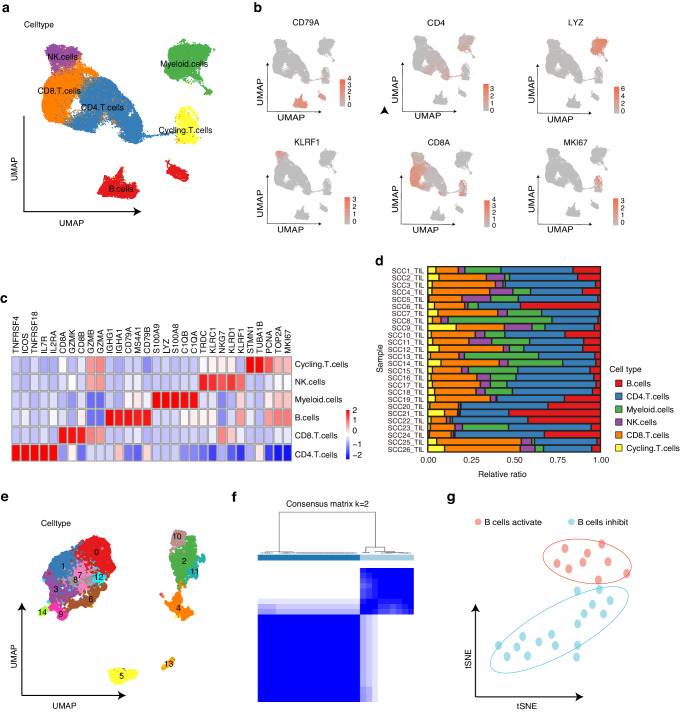
Identification of immunological subtypes based on TIL-Bs by single cell analysis. **a** UMAP of tumor infiltrating immune cells (*n* = 105.943 cells) from 26 samples in HNSCC scRNA-seq, colored by cell clusters. **b** UMAP plots showing expression of classical marker genes from immune cell clusters. **c** Heatmap of signature genes for immune cell clusters. Each cell cluster is represented by five specifically expressed genes. **d** Bar plot showing the immune cell type proportion in each HNSCC sample. **e** UMAP plot showing 15 B cells clusters. **f** Two subgroups were identified using ConsensusClusterPlus R package based on the marker genes. **g** t-SNE plot of HNSCC scRNA-seq cohort (*n* = 26), colored by sample groups

After quality control and data filtering, we obtained single-cell transcriptomes of 105943 immune cells from 26 HNSCC samples (GSE139324). To identify the major populations and subpopulation compositions of tumor infiltrating immune cells, we performed clustering using Seurat to identify the major immune cell types, including NK cells, CD4^+^ T cells, CD8^+^ T cells, cycling T cells, myeloid cells, and B cells (Fig. 1a). Each cell type was identified based on classical marker genes and literature evidence (Fig. 1b, c). The immune cell cluster composition in each patient sample was also shown to observe the state of tumor immune environment in all samples (Fig. 1d).

To interrogate the functional subpopulation and potential role of TIL-Bs in HNSCC, cells defined as B cells in the first level of clustering were selected and re-clustered to identify 15 different B cell subpopulations (Fig. 1e). Then we used Findallmarkers function on these 15 B cell clusters to obtain the B cell-associated differentially expressed genes set (Methods). As a result, a total of 440 marker genes were found, which were used to perform a new unsupervised clustering of the HNSCC samples using ConsensusClusterPlus for feature selection and re-cluster the 25 HNSCC samples (Fig. 1f and Supplementary Fig. 1a). Principal component analysis also classifies the samples into two groups based on the geneset, called the B cells activation group and the B cells inhibition group (Fig. 1g). Samples in these two groups had the largest difference in the proportion and number of their CD8^+^ T cells (Supplementary Fig. 1b–d), indicating a possible distinction in their anti-tumor immunity between these two groups.

**Fig. 2:**
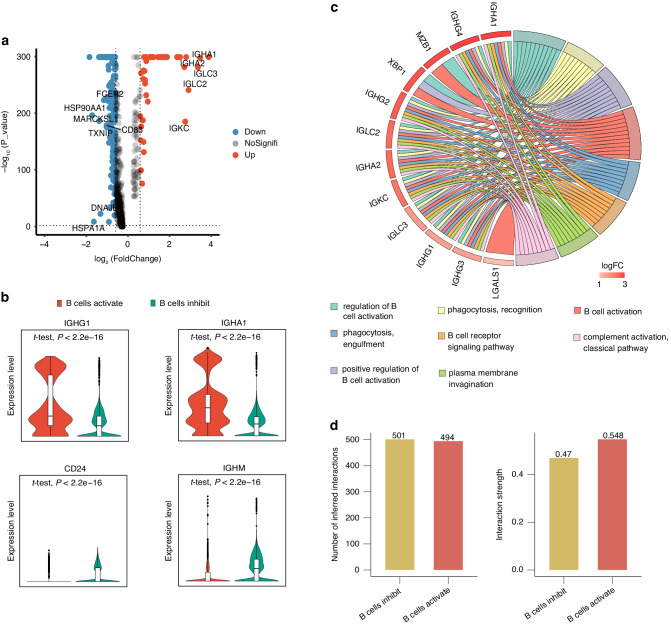
Characterization of immunological subtypes based on TIL-Bs. **a** Volcano plot showing differentially expressed genes between B cells activation group and the B cells inhibition group B cells. Down, downregulated DEGs. NoSignifi, not significant. Up, upregulated DEGs. **b** Violin plot showing expression of IGHG1, IGHA1, CD24 and IGHM in B cells activation group and the B cells inhibition group. **c** GO cluster plot showing a chord dendrogram of the clustering of the expression spectrum of significantly upregulated genes in B cells based on the B-cell signature genes classification. **d** The differential number of interactions (left) or interaction strength (right) in the cell-cell communication network between the two groups was shown by the circle plots

Previous data revealed disparities in tumor immunity based on single-cell sample groupings of B-cell signature genes, therefore we dissected the discrepancies between the two groups at the genetic level. As indicated in the volcano map, using log2FC > 1 and *P* < 0.05 as cut-off thresholds, we identified 43 differentially expressed genes (21 up-regulated and 22 down-regulated genes) between the B cell activation and B cell inhibition groups (Fig. 2a). We defined the 21 upregulated genes as B cell activation gene signature (BCAGS) and used this signature for the next validation analysis. Most of the up-regulated genes were associated with activation of B cells, whereas the down-regulated genes were associated with inhibition of B cells (Fig. 2a, b). The up-regulated genes were highly enriched in the regulation of B cell activation, B cell receptor signaling pathway, and B cell activation pathway, according to GO function enrichment analysis (Fig. 2c). We then performed GO function enrichment for DEGs in other immune cell clusters in these two groups, and found that the upregulated genes in the B cells activation group facilitated multifunctional immunological regulation, such as positive regulation of leukocyte activation, antigen binding, cell killing and immune cell recruitment (Supplementary Fig. 2a–e).

Emerging evidence had demonstrated immune cell function or communication was skewed in malignancies. However, a global profile of immune cell communication in HNSCC based on this grouping approach is largely undefined. To systematically investigate cell-cell communication in the B-cell activation and B-cell suppression groups, we used CellChat to conduct an unbiased analysis of the overall and differential number and strength of interactions, as well as “ligand-receptor” interactions. Intriguingly, the strength of cell-cell communication activation differed between the two groups. As shown, the overall strength of immune cell interactions increased in the B cells activation group (Fig. 2d), and T-cell direct interactions were more active (Supplementary Fig. 2f). And in the ligand-receptor interaction, the B cells activation group exhibited more CSF, CXCL, and MHC-I signaling pathway, while CD22, and CD23 was inhibited, indicating that the B cells activation group improved anti-tumor immunity through cell-cell intercommunication (Supplementary Fig. 2g). These cell-cell communication data provide evidence for the formation of immune cell-cell communication stratification based on the B-cell signature gene HNSCC classification.

### Validation for B-cell classification suggests the consistency in different scRNA-seq datasets

**Fig. 3:**
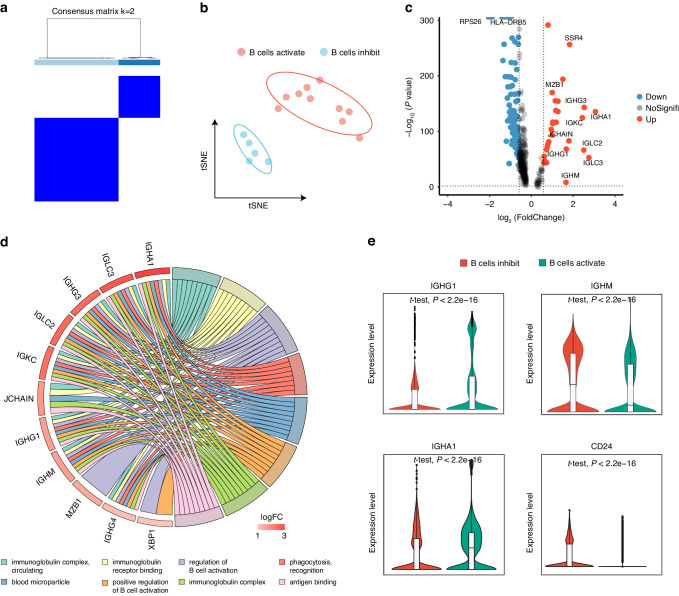
Validation of classification method based on TIL-Bs in another dataset. **a** Two subgroups were identified using ConsensusClusterPlus R package based on the B-cell signature gene. **b** t-SNE plot of HNSCC scRNA-seq cohort (*n* = 15), colored by sample groups. **c** Volcano plot showing differentially expressed genes between B cells activation group and the B cells inhibition group B cells. Down, down regulated DEGs. NoSignifi, not significant. Up, up regulated DEGs. **d** GO cluster plot showing a chord dendrogram of the clustering of the expression spectrum of significantly upregulated genes in B cells based on the B-cell signature genes classification. **e** Violin plot showing expression of IGHG1, IGHA1, CD24 and IGHM in B cells activation group and the B cells inhibition group

To further demonstrate the merits of the obtained B-cell signature genes for classifying HNSCC patients, we collected another HNSCC single-cell data (GSE164690) as the validation cohort. We selected tumor-infiltrating immune cell populations and defined them according to classical markers or evidence from the literature, including NK cells, CD4^+^ T cells, CD8^+^ T cells, myeloid cells, and B cells (Supplementary Fig. 3a–c). The percentage of immune population cells is also listed for all 18 patients (Supplementary Fig. 3d). We also performed unsupervised clustering of these 18 samples using BCAGS obtained previously, using ConsensusClusterPlus for feature selection and re-clustering (Fig. 3a). Consistently, the patients were clustered into two distinct groups in this cohort (Fig. 3b). Furthermore, in the grouping based on the B-cell signature gene set, we also observed differences in the number and proportion of CD4^+^ T cells, CD8^+^ T cells, and B cells between the two groups (Supplementary Fig. 3e, f), which supports the possibility that there is a gap in anti-tumor immunity with this classifying approach.

We also verified the consistency of this classifying in the single-cell cohort at the gene level. Volcano plots show the DEGs in the transcriptome level between the B cells activation and B cells inhibition groups, where 29 up-regulated and 101 down-regulated genes were observed (Fig. 3c). GO function enrichment showed the upregulated DEGs were significantly enriched in the immunoglobulin complex, circulating, immunoglobulin receptor binding, and regulation of B cell activation pathway (Fig. 3d). Consistent with the previous cohort, the up-regulated genes were mostly B-cell activation-related genes (Fig. 3e). We also performed GO function enrichment on the DEGs of other immune cell populations in these two groups and showed that the upregulated genes in the B cells activation group mediated multifunctional immune regulation, such as positive T cell selection, positive regulation of leukocyte activation, lymphocyte-mediated immunity, and MHC class II protein complex (Supplementary Fig. 4a–e). The above results suggest that the classifying method is consistent across different single-cell cohorts.

Furthermore, there was a high degree of consistency for cell-cell communication in both groups, with enhanced total number and strength of immune cell interactions in B cells activation group (Supplementary Fig. 4e) and more active direct T-cell population interactions (Supplementary Fig. 4f). And in ligand-receptor interaction, the B cells activation group showed more active CSF, CXCL, and MHC-I signaling pathway, while CD22 was inhibited, indicating that the B cells activation group improved anti-tumor immunity through cell-cell interactions (Supplementary Fig. 4g). These cell-cell communication data provide evidence for consistency in different single-cell cohorts based on the B-cell signature gene classifying of HNSCC.

### B-cell classification in TCGA HNSCC and association with overall survival

**Fig. 4:**
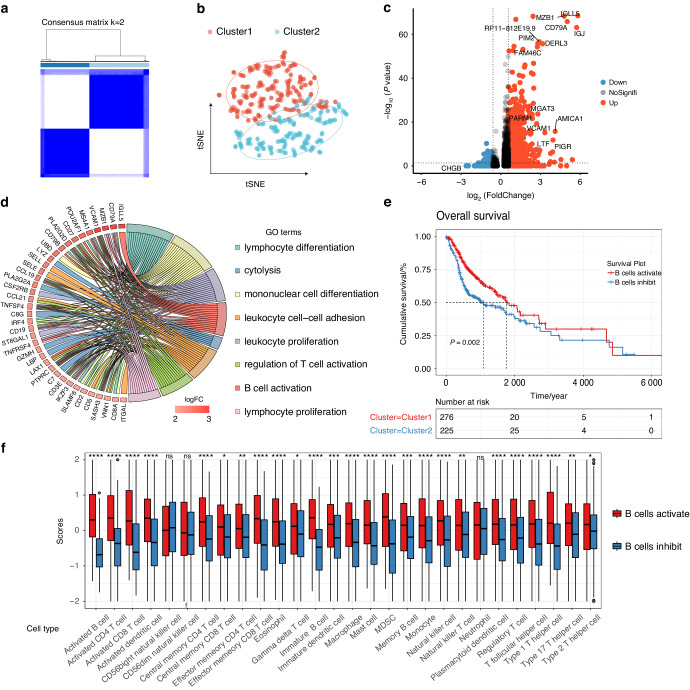
Application of classification method based on TIL-Bs in TCGA cohort. **a** Consensus matrix of TCGA HNSCC cohort (*n* = 501). **b** t-SNE plot shows TCGA HNSCC samples were divided into two clusters. **c** Volcano plot depicts the differentially expressed genes between patients from cluster 1 and cluster 2. **d** GO cluster plot showing a chord dendrogram of the clustering of the expression spectrum of significantly upregulated genes in B cells activation group based on the B-cell signature genes classification. **e** The Kaplan-Meier overall survival curves of TCGA HNSCC patients between B cells activation group and the B cells inhibition group. TCGA samples were stratified by the B-cell signature genes. **f** The enrichment levels of 28 immune-related cells (by ssGSEA analysis) and types in the B cells activation group and B cells inhibition group

To verify whether this B-cell signature gene classifying method applicable to TCGA HNSCC, we performed unsupervised clustering of RAN-seq expression profiles of 501 patient samples and obtained two clusters based on BCAGS (Fig. 4a), and tSNE clustering showed that the samples divided into two groups (Fig. 4b). As shown in the volcano plot, 388 upregulated genes and 42 downregulated genes were identified in the two groups from TCGA HNSCC patients (Fig. 4c). GO function enrichment analysis for the DEGs in the B cells activation group were found to be significantly enriched in lymphocyte activation and immune activation related pathways (Fig. 4d). We correlated the grouping with clinical information to explore the prognostic value of this tumor typing. Previous studies have shown that B cell is associated with improved overall survival in tumor patients.^21,22^ In our study, the Kaplan-Meier curve showed that patients in the B cells activation group had a significant better survival prognosis (Fig. 4e).

To explore the effect of the classifying method on the infiltration fraction of immune cells, ssGSEA was used to visualize the relative abundance of 28 infiltrating immune cell populations. B cells activation group regions showed a higher abundance of immune cell infiltration (Fig. 4f and Supplementary Fig. 5a). Pearson’s correlation study revealed that the abundances of these two groups were positively related within a local environment (Supplementary Fig. 5b). Samples from the B cells activation group in TCGA HNSCC had a higher proportion of tumor-infiltrating lymphocytes cells (Supplementary Fig. 5c), than the B cells inhibition group, as estimated by CIBERSORT. Taken together, our data demonstrated the consistency of such classifying method both in single-cell and RNA-seq cohort.

### B-cell classification accurately predict immunotherapy response

**Fig. 5:**
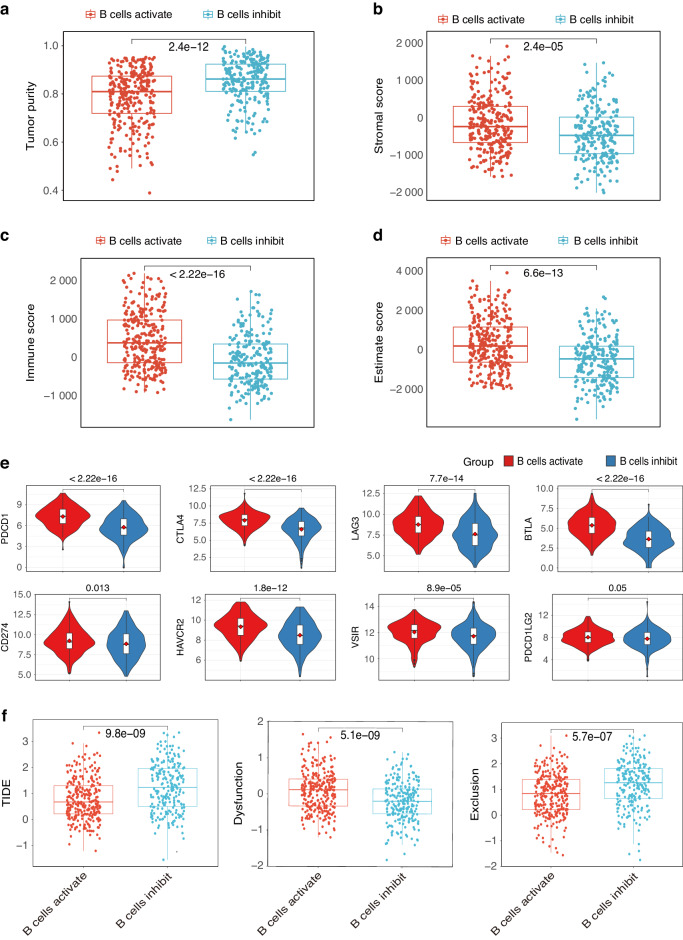
B cells-based classification predicts immunotherapy response accurately. Box plots showing lower tumor purity (**a**), and higher stromal score (**b**), immune score (**c**), and ESTIMATE score (**d**) in the B-cell activation group. **e** Box plots shows the different expression levels of the typical immune inhibitory receptors (PDCD1, CTLA4, LAG3, BTLA, CD274, HAVCR2, VSIR, and PDCD1LG2) between two groups. **f** Patients in the B cells activation group showed lower TIDE score, higher Dysfunction, and higher Exclusion score

We also assessed the infiltration fraction of stromal and immune cells in the tumor using the ESTIMATE function and inferred the purity of the tumor. Higher immune scores, stromal scores, and ESTIMATE score in the B cells activation group, lower tumor purity than in the B cells inhibition group were observed (Fig. 5a–d). Our previous data suggest the presence of a higher abundance of immune cells in the B cells activation group, indicating a potential greater susceptibility to immunotherapy. Meanwhile, we explore the expression profile analysis of typical immune inhibitory molecules (PD-1, CTLA4, LAG3, BTLA, CD274, HAVCR2, VSIR, and PDCD1LG2) on the two groups. Patients within B cells inhibition group showed significant down-regulated inhibitory receptors (Fig. 5e). To further explore possible correction between the classification and immunotherapy response, we calculate the tumor immune dysfunction and exclusion (TIDE) scores in both groups to assess the potential for tumor immune evasion and to predict response to immune response. The results showed B cells activation group had lower TIDE scores than B cells inhibition group (Fig. 5f). The lower TIDE score is usually correlated with better ICB therapy. Our results indicated that patients in the B cells activation group were possibly sensitive to the ICB therapy.

### Four-gene prognostic model development and validation in HNSCC

**Fig. 6:**
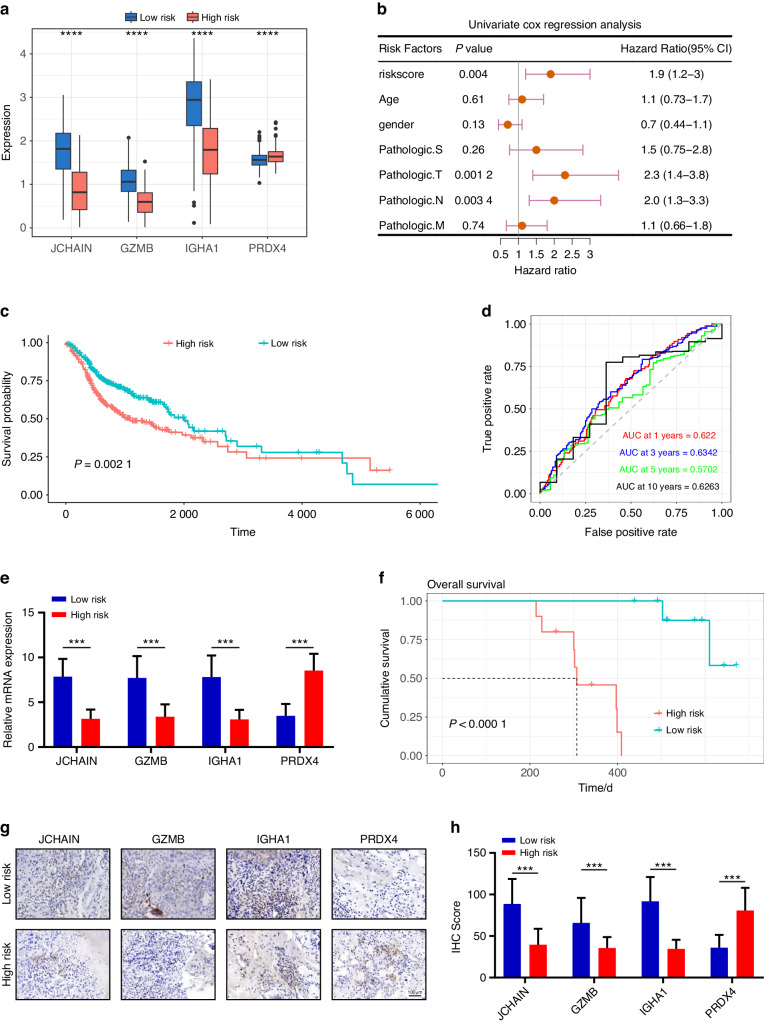
Unveiling a four-gene risk model for prognostication in HNSCC patients. **a** Comparative boxplot delineating the modeling-related gene expression levels within the two groups. **b** Forest plot mapping out OS-related clinical factors via single-factor Cox regression analysis. **c** Kaplan–Meier survival plots manifesting prognosis-linked risk scores’ impact. **d** ROC curve spectra, a predictive gauge for 1, 3, 5, and 10-year OS dynamics. **e** Comparative analysis spotlighting differential gene expressions between high and low-risk groups. **f** Kaplan-Meier survival curves of HNSCC patients based on four genes Risk-scores. Representative IHC staining images (**g**) and IHC score (**h**) in the two groups. Scale bar, 100 μm

Our investigation utilized LASSO regression analysis to optimize the BCAGS, unveiling a four-gene ensemble (JCHAIN, GZMB, IGHA1, and PDRX4), fostering the construction of a pivotal prognostic risk model. Among these, JCHAIN, GZMB, and IGHA1 showed significantly elevated expression in the low-risk group, while PDRX4 demonstrated notably increased expression in the high-risk group (Fig. 6a). Subsequently, employing univariate Cox regression analyses across the TCGA cohorts, we sought to discern whether the risk score, untethered from conventional clinical factors, could autonomously prognosticate patient outcomes. Encouragingly, our findings unveiled the risk score (HR: 1.9; 95% CI: 1.2–3) as an autonomous harbinger of OS (Fig. 6b). Notably, KM and log-rank analyses illuminated the stark divergence in OS between high and low-risk HNSCC cohorts (Fig. 6c). Moreover, the efficacy of risk scores in forecasting OS within the TCGA cohort was conspicuous (AUC for 1-, 3-, 5-, and 10-year OS: 0.622 0, 0.634 2, 0.570 2, and 0.626 3, respectively), showcasing their adeptness in long-term prognostication (Fig. 6d). To fortify the prognostic validity of our model, we enlisted a clinical cohort comprising 20 heterogeneous HNSCC patients, aiming to authenticate the expression patterns of the quartet genes (JCHAIN, GZMB, IGHA1, and PRDX4). Employing mRNA quantification of these genes via RT-qPCR, we delineated a precise demarcation between low and high-risk patients within our clinical cohort based on the genes’ expression levels. Strikingly, the JCHAIN, GZMB, and IGHA1 exhibited markedly escalated expression within the low-risk category, while PRDX4 showed a notable decrease (Fig. 6e). Subsequent Kaplan-Meier analysis underscored a significant correlation between the high risk group and a deteriorated prognosis in HNSCC patients (Fig. 6f). Complementary to RT-qPCR, our investigative stride delved deeper through immunohistochemistry (IHC) analysis, unraveling spatial and protein-level insights that corroborated the expression pattern of these genes within the high-risk stratum (Figs. 6g, h). This substantiation further bolsters the nexus between these genes and adverse clinical outcomes. Remarkably, our experimental results seamlessly aligned with our constructed risk model, affirming the potential of these four genes as robust prognostic biomarkers in predicting survival outcomes among HNSCC patients.

## Discussion

With the in-depth research on tumor immune microenvironment, anti-tumor immunotherapy, which aims to activate autoimmunity, has received more attention, and become the fourth treatment method alongside surgery, radiotherapy, and chemotherapy.^23,24^ B cells are an important component of adaptive immunity, and although the focus of tumor immunology has been T cells, emerging studies have shown that B cells infiltration in tumors is associated with better patient survival,^17,25^ suggesting a great potential for effective activation of B cell immune function in antitumor immunity. However, studies on B-cell immune function-based classifying in HNSCC remain unknown.

The scRNA-seq technique has emerged as an available tool for analyzing the molecular feature of immune cell infiltration in TME. In this study, we collected all tumor-infiltrating immune cells from the HNSCC scRNA-seq data and defined them as CD4^+^ T cells, CD8^+^ T cells, NK cells, circulating T cells, myeloid cells, and B cells. In addition, we isolated B-cell subpopulations, identified critical differential expressed gene sets associated with B-cell immune function activation or suppression, and classified HNSCC patient samples based on this gene set as B-cell activated group and B-cell suppressed group, respectively. Similarly, researchers performed molecular typing of colorectal cancer based on differential expressed gene, DNA copy number, and gene regulatory networks.^26^ Another study, based on an artificial intelligence learning framework, classified 10 cell states of tumors with more significant predictive implications for tumor prognosis.^27^ Differential expressed genes between the two typed B cells showed that the B cells activation group upregulated the expression of genes associated with B cell activation. Further GO functional analysis revealed that the upregulated genes were significantly enriched in pathways such as immune activation in the tumor immune microenvironment. And our cell-cell communication analyses predicted an increased interactions between the tumor-infiltrating immune cells in the B cells activation group. It is also consistent with the roles B cells play in antitumor immunity, where they can impede tumor development by secreting immunoglobulins and directly killing cancer cells, as well as promote tumor death by other immune cells.^17,25,28^ High activity of macrophage chemotactic pathways was also detected in plasma cells, for example, suggesting a potential role of B cells in promoting the recruitment of macrophages to TME in NPC.^29^ Of note, we then validated the classification performance of the B cells DEGs signature in another cohort of HNSCC single-cell data, demonstrating the applicability of this approach in different cohorts.

Although in this work, HNSCC classification based on B cells DEGs was derived from scRNA-seq data, it can be generalized to any scRNA-seq samples and RNA-seq samples with suitable expression data. Thus, the typing method based on the B cells DEGs was employed in TCGA HNSCC cohort. Intriguingly, our classification method divided patients into groups with significant OS stratification. This shows that we can classify HNSCC into two distinct tumor groups based on B cells infiltration, which aids in HNSCC for various immunotherapies. Meanwhile, given the prognostic relevance of tumor microenvironment, we compared the differences in immune infiltration between the two groups of patients. The results showed that the proportion of lymphocytes and immune infiltration score was larger in the B cells activation group of patients, which is consistent with earlier data.

One of the hallmarks of tumor cells is the ability to adapt or evade surveillance by the immune system.^30–32^ The potency of this ability of tumor cells makes the response to immune checkpoint inhibitors vary from patients. And the involvement of B cells in the tumor microenvironment, as an essential part of the immune system, impacts the fate of the immune response. Currently, the most extensively utilized predictors of PD-1/PD-L1 inhibitor response are frequently single gene expressions, which are insufficient to accurately predict ICB results. TIDE is a newly discovered immunotherapy predictor that has been demonstrated to outperform other biomarkers or indicators.^33–35^ We explored the association between grouping and the TIDE signature to show that the classification based on B cells DEGs signature may be employed as a biomarker of immunotherapeutic response. Results showed that patients in the B cells activation group had lower TIDE and Dysfunction scores, suggesting that tumors with B cells activation group had strong immunogenicity to activate immune cells to detect cancers. Higher typical immune inhibitory receptors expression in B cells activation group was observed, indicating decreased anti-tumor immunity in patients.

Previous research has highlighted the effectiveness of gene signatures in guiding both cancer treatments and prognostic assessments.^36,37^ Through the utilization of sophisticated LASSO and random forest survival models, we identified four robust genes (JCHAIN, GZMB, IGHA1, and PDRX4) that exhibit significant potential in predicting overall patient survival, exemplified by the AUC values. Our innovative predictive model’s meticulous biomarker assessment enables the stratification of patients based on risk, thereby facilitating the tailoring of treatment strategies. This empowers high-risk patients to benefit from vigilant monitoring, novel interventions, or the opportunity to participate in clinical trials, while offering low-risk patients the chance to avoid unnecessary aggressive treatments. The integration of our model into routine assessments offers a comprehensive outlook on prognosis, empowering informed decision-making and optimizing treatment modalities to elevate patient outcomes significantly. This amalgamation between research findings and practical applications underscores the transformative potential of our model in revolutionizing cancer prognosis, ultimately prioritizing the well-being of patients. Recognizing the imperative necessity to bridge the gap between research discoveries and their practical implementation, our aim is to elucidate the effective integration of our prognostic prediction model into clinical practice. The reliance on single-cell RNA sequencing data from the GEO may not fully represent the diverse HNSCC population. Additionally, the unsupervised clustering approach used for classifying HNSCC samples could introduce subjective biases. While validation with TCGA data strengthens the findings, further external validation is necessary. Moreover, the patient selection and sample characteristics from GEO and TCGA might affect the outcomes, and the proposed prognostic model and biomarkers require validation in broader cohorts. These points highlight the need for careful interpretation and further research to substantiate the findings.

In conclusion, we established and validated a B cells DEGs signature-based HNSCC classification by combined analysis of single-cell and TCGA HNSCC data, and the classification was effective in predicting patient prognosis and patient response to immunotherapy. Our prognostic risk model could provide guidance for clinical HNSCC classification and immunotherapy. Our study lays the foundation for tumor infiltration-based immune cell tumor classification for the precise treatment of HNSCC.

## Materials and methods

### Data download

Single-cell transcriptome data from two HNSCC cohorts, GSE139324 and GSE164690, were downloaded from Gene Expression Omnibus (GEO) database (http://www.ncbi.nlm.nih.gov/geo/) and used to establish B cell-based HNSCC classification, construct activated B cell genetic signature and to verify the consistency of HNSCC classification. We selected these datasets due to their extensive coverage of B cell clusters, which are essential to the goals of our research. Our intention in using a variety of samples was to reduce possible biases and confirm that our results are applicable across different patient groups. In addition, to explore the ability of the activating B cell gene signature to predict clinical prognosis, we downloaded transcriptomic and clinical data from the TCGA database for 501 HNSCC patients. We also downloaded information on HNSCC patient cohorts from the GEO database for further survival-related genetic screening.

### HNSCC patient samples

Twenty patients undergoing surgery at the Oral and Maxillofacial Surgery Department of the First Affiliated Hospital, Sun Yat-Sen University, provided HNSCC tumor tissue samples. Consent forms were signed by all participants before the study. The Institutional Review Board of Sun Yat-Sen University’s First Affiliated Hospital approved all patient-related research.

### scRNA-seq data processing

We performed scRNA-seq data analysis by using the Seurat 4.0 package in R. Briefly, the Seurat object was created by importing the sample expression matrixes into R using the Read10X function and integrated the relevant clinical information. quality control process involved several steps. Firstly, we chose to retain only those cells that exhibited gene expression counts ranging from 200 to 10 000. Furthermore, we filtered out cells with more than 10% of their reads mapped to the mitochondrial genome, as this can be indicative of poor cell quality. Additionally, we employed measures to remove doublets (cells mistakenly identified as a single cell) from each sample to ensure the purity and accuracy of our dataset. After quality control, we proceeded to identify highly variable genes (HVGs) as these genes are often key in distinguishing between different cell types. We selected the top 2000 HVGs for further analysis. Using these genes, we performed principal component analysis (PCA) to reduce the dimensionality of our dataset and to identify the major axes of variation. Based on the PCA results, we conducted a cluster analysis using the top 1-20 principal components. We primarily identified the following major cell types: B cells, CD4^+^ T cells, CD8^+^ T cells, cycling T cells, and myeloid cells.

### Acquisition of geneset for Consensus Clustering

To explore the role of B cells in HNSCC, we obtained the differentially expressed genes set among all identity B cell subsets. In brief, the Findallmarkers function was applied to identity Bcells-realated subsets to calculate the differentially expressed genes (DEGs) from each cell subpopulation with a threshold of Log2FC > 1, *P* < 0.05. This geneset which is then used for consensus clustering contains all possible characteristic genes of the B-cell subpopulation.

### BCAGS identification

To obtain the precise set of characteristic genes representing B-cell subpopulations, DEGs of B-cell pseudo-bulk were identified using the “limma” method. 22 genes in DEGs of B cell activation group (Log_2_FC > 1, *P* < 0.05) were selected out for building B cell activation gene signature (BCAGS).

### GO enrichment analysis

To probe BCAGS at the genetic level, Gene ontology (GO) enrichment analysis of B cell activation group was performed using the “clusterProfiler” R package. We focused on Homo sapiens as the selected species and considered biological processes with a p-value threshold of <0.05. The results of GO enrichment analysis of B cell activation group showed that the upregulated genes were enriched in immune cell activation-related pathways.

### ssGSEA

The single-sample gene set enrichment analysis (ssGSEA) was introduced to quantify the relative infiltration of 28 immune-related cell types in the tumor microenvironment of two different subgroups. Feature gene panels for each immune cell type were obtained from a recent publication. The relative abundance of each immune cell type was represented by an enrichment score in ssGSEA analysis. The ssGSEA score was normalized to unity distribution, for which zero is the minimal and one is the maximal score for each immune cell type, and the histograms were based on normalized ssGSEA scores. The bio-similarity of the immune cell filtration was estimated by multidimensional scaling (MDS) and a Gaussian fitting model.

### CIBERSORT

In this study, CIBERSORT was used to assess differences in the abundance of immune infiltrates based on BCAGS subgroups in HNSCC cohorts from the TCGA and GEO databases. The scores of each immune cell subpopulation were quantified by the “CIBERSORT” R package based on the transcriptomic data of HNSCC. All immune cell types in different subgroups were predicted by the leukocyte signature matrix (LM22) of CIBERSORT.

### Cell communication

The “CellChat” R package is used for cell-cell communication analysis and creates CellChat objects based on two sets (BCSGS based groups) of gene expression matrixes. The mergeCellChat function was used to merge the two groups of CellChat objects and analyze the differences in the number of inferred interactions and the strength of interactions. And netVisual_diffInteraction was utilized to visualize the number of inferred interactions and the strength of interactions. Cell-cell communications between the two groups in terms of “secreted signalling” and “ receptor-ligand” were compared in different immune cell populations.

### Establishment of BCAGS prognostic risk model

To construct a prognostic risk model, TCGA HNSCC cases were meticulously analyzed. Utilizing the “glmnet” package in R, a rigorous least absolute shrinkage and selection operator (LASSO) regression was conducted to identify genes most pertinent to prognosis within the BCAGS. Subsequently, employing the “forest plot” package, univariate Cox regression analyses were performed, defining genes with hazard ratios (HR) > 1 as risk factors and HR < 1 as protective factor. Individualized risk scores were computed for patients as follows: risk score = h0*e^∑ i = 0nexp () and stratified survival curves plotted via the Kaplan–Meier method. Uni-cox analyses further explored the intricate relationship between risk scores, clinicopathological characteristics, and overall survival (OS) in HNSCC.

### Real-time quantitative PCR

Tissue samples obtained from patients diagnosed with HNSCC were subjected to RNA extraction using the TRIzol reagent (ThermoFisher, 15596018). The RNA concentration and purity were meticulously assessed using the NanoDrop 2000 spectrophotometer. Subsequently, RNA was reverse transcribed into complementary DNA (cDNA) utilizing the Vazyme Reverse Transcription kit (R312-01) following standardized protocols. Real-time PCR was then performed on 1 μg of cDNA using the SYBR Green™ Master Mix (AG, AG11701). Technical replicates for each sample were conducted three times. GAPDH was employed as the reference gene to accurately quantify the target mRNA levels. The primer pairs used for Real-time PCR amplification were as follows:

*JCHAIN* (forward: 5′-CCAGGATCATCCGTTCTTCCGA-3′; reverse: 5′- CAAATCTGGTTCTCAATGGTGAGG-3′).

*GZMB* (forward: 5′-CCCTGGGAAAACACTCACACA-3′; reverse: 5′- GCACAACTCAATGGTACTGTCG-3′).

*IGHA1* (forward: 5′-GCAGCATTCGGATTCACATTC-3′; reverse: 5′- GATGTTCCTGATGTTGTCTCTGG-3′).

*PRDX4* (forward: 5′-CGCTTTTGGCGACAGACTTGAAG-3′; reverse: 5′- CCAAGTCCTCCTTGTCTTCGAG-3′).

*GAPDH* (forward: 5′-AGATCCCTCCAAAATCAAGTGG-3′; reverse: 5′- GGCAGAGATGATGACCCTTTT-3′).

### IHC staining

The tissue specimens were initially fixed in 10% buffered formalin, preserved in 70% ethanol, followed by embedding in liquid paraffin, and subjecting to permeabilization treatments at 65 °C. Then the processed paraffin blocks were finely sectioned into 5 μm slices, deparaffinized in 4 °C ethanol, and rehydrated in phosphate-buffered saline (PBS) supplemented with 5% bovine serum albumin (BSA) to prevent non-specific binding. Following an overnight incubation with primary antibodies JCHAIN (Affinity Biosciences, DF14810, 1:200), GZMB (Abcam, ab237847, 1:200), IGHA1 (Abnova, H00003493-W01P, 1:200), and PDRX4 (Affinity Biosciences, DF6425, 1:200), sections underwent a 30-minute incubation with HRP-secondary antibodies. Finally, sections were stained with DAB solution, counterstained with neutral background reagent, and observed microscopically. Image analysis software was employed for thorough section analysis and to obtain IHC score.

### Survival analysis

After classifying the HNSCC using the B cell subsets gene signature, each tumor group was associated with patient overall survival. Specifically, patient samples from the TCGA and GEO databases were clustered into two groups, Cluster1 and Cluster2, using the obtained B cell subsets gene signature consisting of 43 genes, respectively. Kaplan-Meier survival curves for the two groups were generated by the “survivor” R package, and survival was considered significant by log-rank test *P* < 0.05.

### Statistical analysis

In this study, the researchers were blinded to clinical annotations in defining cell types and cell subtypes. Student’s *t*-test and Wilcoxon signed-rank test were used to compare differences between groups, and all statistics were considered statistically different at *P* < 0.05. R software (version 4.0.3) was used to perform data processing and analysis. All parameter settings of the R package used in this study were default values, except where noted.

### Supplementary information

supplementary information
