# Supplementary material for: Single cell analysis unveils B cell-dominated immune subtypes in HNSCC for enhanced prognostic and therapeutic stratification
Source: Int J Oral Sci. 2024 Apr 16;16:29. doi: 10.1038/s41368-024-00292-1 (PMC11018606; doi:10.1038/s41368-024-00292-1)
Supplement: Supplementary file 1 — supplementary information [file 41368_2024_292_MOESM1_ESM.docx]

**Supplementary information**

**Single cell analysis unveils B cell-dominated immune subtypes in HNSCC for enhanced prognostic and therapeutic stratification**

Kang Li^1*^, Caihua Zhang^1*^, Ruoxing Zhou^1*^, Maosheng Cheng^1^, Rongsong Ling^2^, Gan Xiong^3^, Jieyi Ma^1^, Yan Zhu^1^, Shuang Chen^1^, Jie Chen^3#^, Demeng Chen^1#^, Liang Peng^4#^

1. State Key Laboratory of Oncology in South China, Department of Oral and Maxillofacial Surgery; Institute of Precision Medicine; Center for Translational Medicine, The First Affiliated Hospital of Sun Yat-sen University, Guangzhou 510080, China.
2. Institute for Advanced Study, Shenzhen University, Shenzhen, 518057, China.
3. Hospital of Stomatology, Guangdong Provincial Key Laboratory of Stomatology, Guanghua School of Stomatology, Sun Yat-Sen University, Guangzhou 510055, China.
4. Senior Department of Oncology, the Fifth Medical Center of PLA General Hospital, NO.8 the east street, Fengtai District, Beijing, 100071, China

# Corresponding Author. Jie Chen, [chenj827@mail.sysu.edu.cn](mailto:chenj827@mail.sysu.edu.cn)

# Corresponding Author. Demeng Chen, [chendm29@mail.sysu.edu.cn](mailto:chendm29@mail.sysu.edu.cn)

# Corresponding Author. Liang Peng, [pengliang_301@163.com](mailto:pengliang_301@163.com).

*These authors contribute equally to this work.


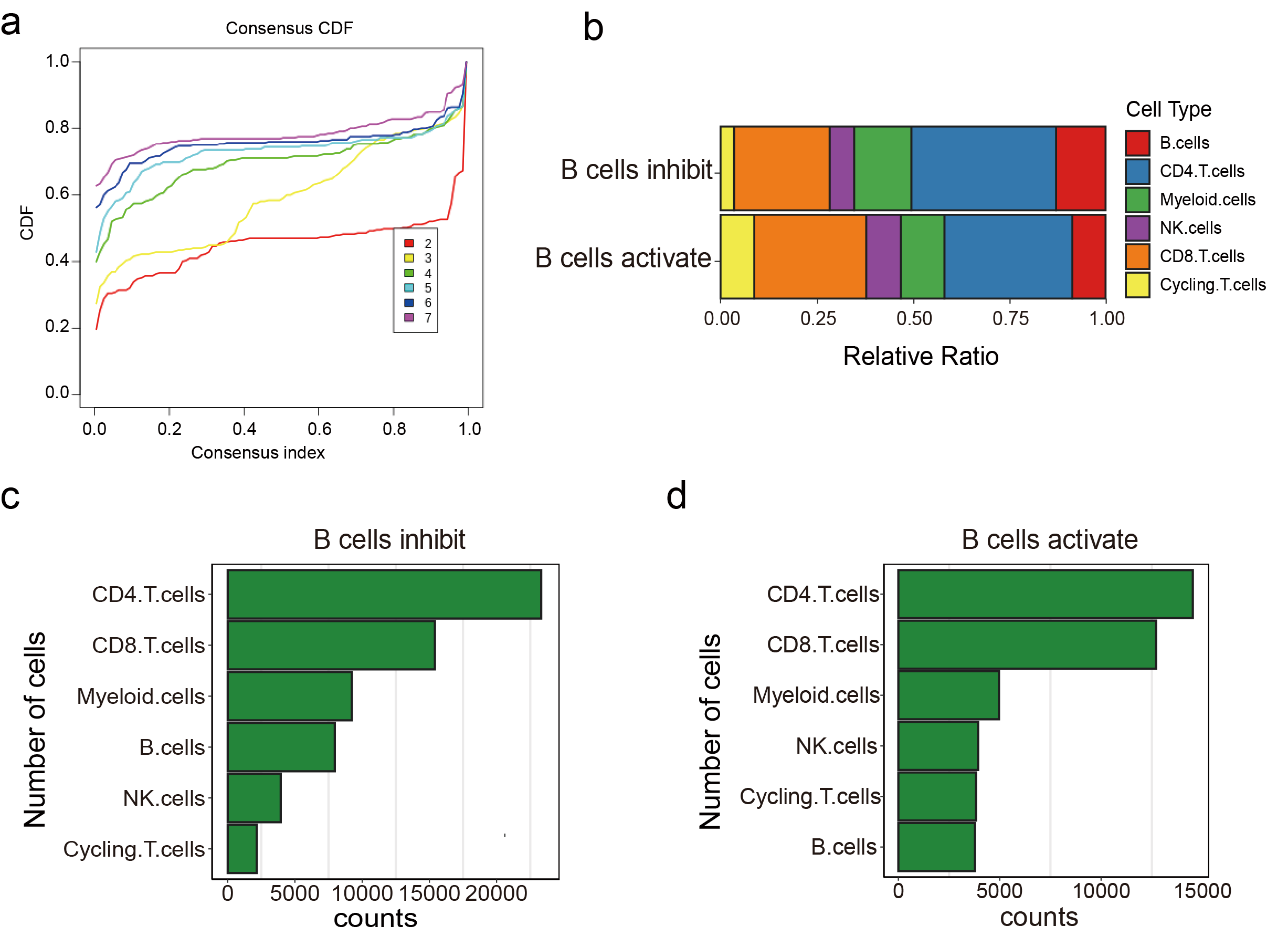


**Figure S1 Quantitative differences between the two immune subtypes**

a. Consensus cumulative distribution function (CDF) plots for 2–7 clusters.

b. Bar plot showing the immune cell type proportion in B cells activation group and the B cells inhibition group.

c. and d. Bar plots of immune cell clusters cell number in B cells activation group (c) and the B cells inhibition group (d).


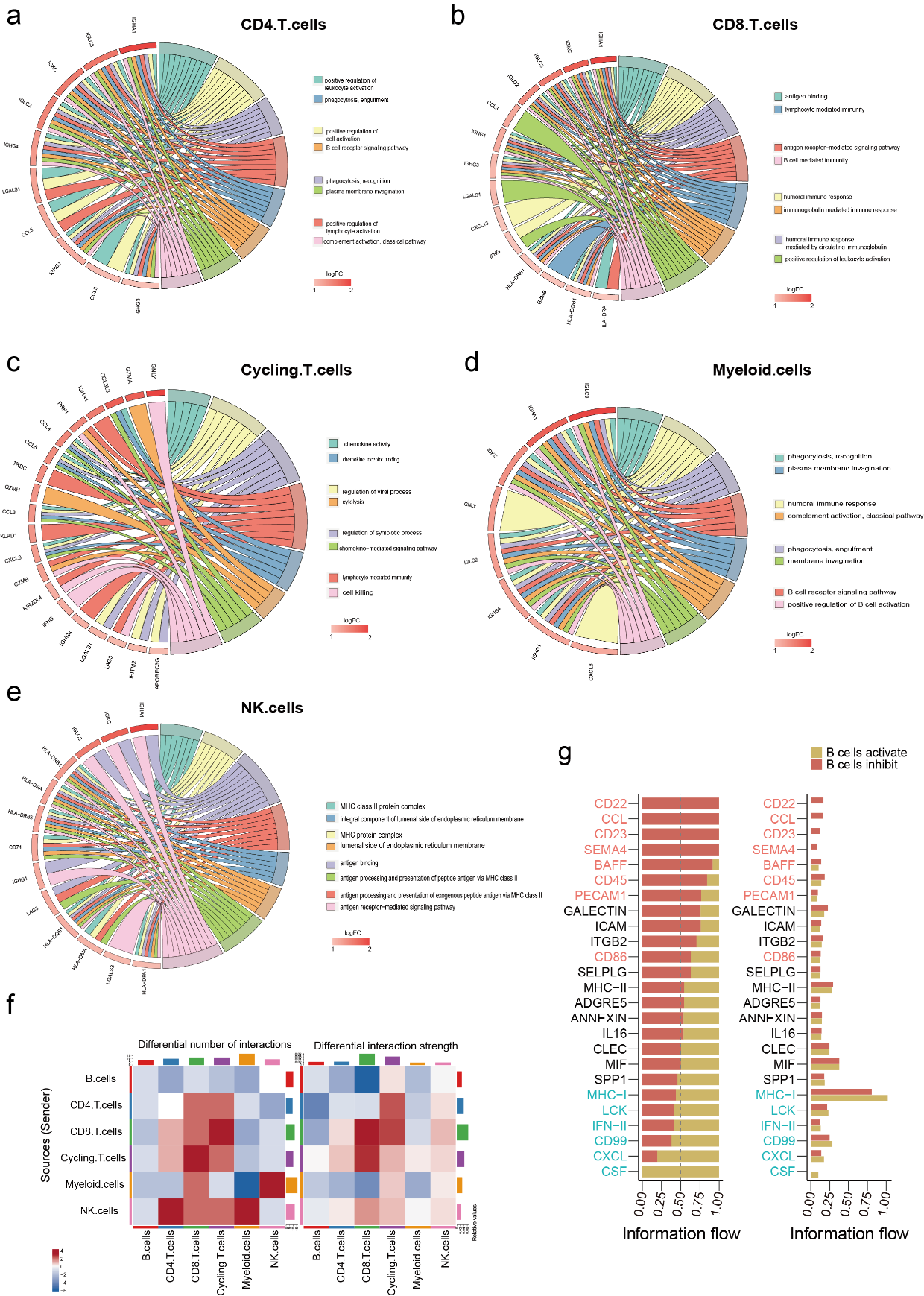


**Figure S2 GO and interaction** **analysis of immune cells in B cells activation group**

a-e. GO cluster plot showing a chord dendrogram of the clustering of the expression spectrum of significantly upregulated genes in CD4^+^ T cells (a), CD8^+^ T cells (b), cycling T cells (c), myeloid cells (d), and NK cells (e) based on the B-cell signature genes classification.

f. Heatmap showing the differential number of interactions (left) and differential interaction strength (right) in immune clusters between the two groups. The red to dark blue gradient indicates the cell-cell communication weight values from high to low in the B cells activation group in the heatmap.

g. Bar plot showing differences in ligand-receptor interaction signaling pathways between the two groups. Signaling pathways colored in red were more enriched in B cells inhibition group, pathways colored in brown are more enriched in B cells activation group.


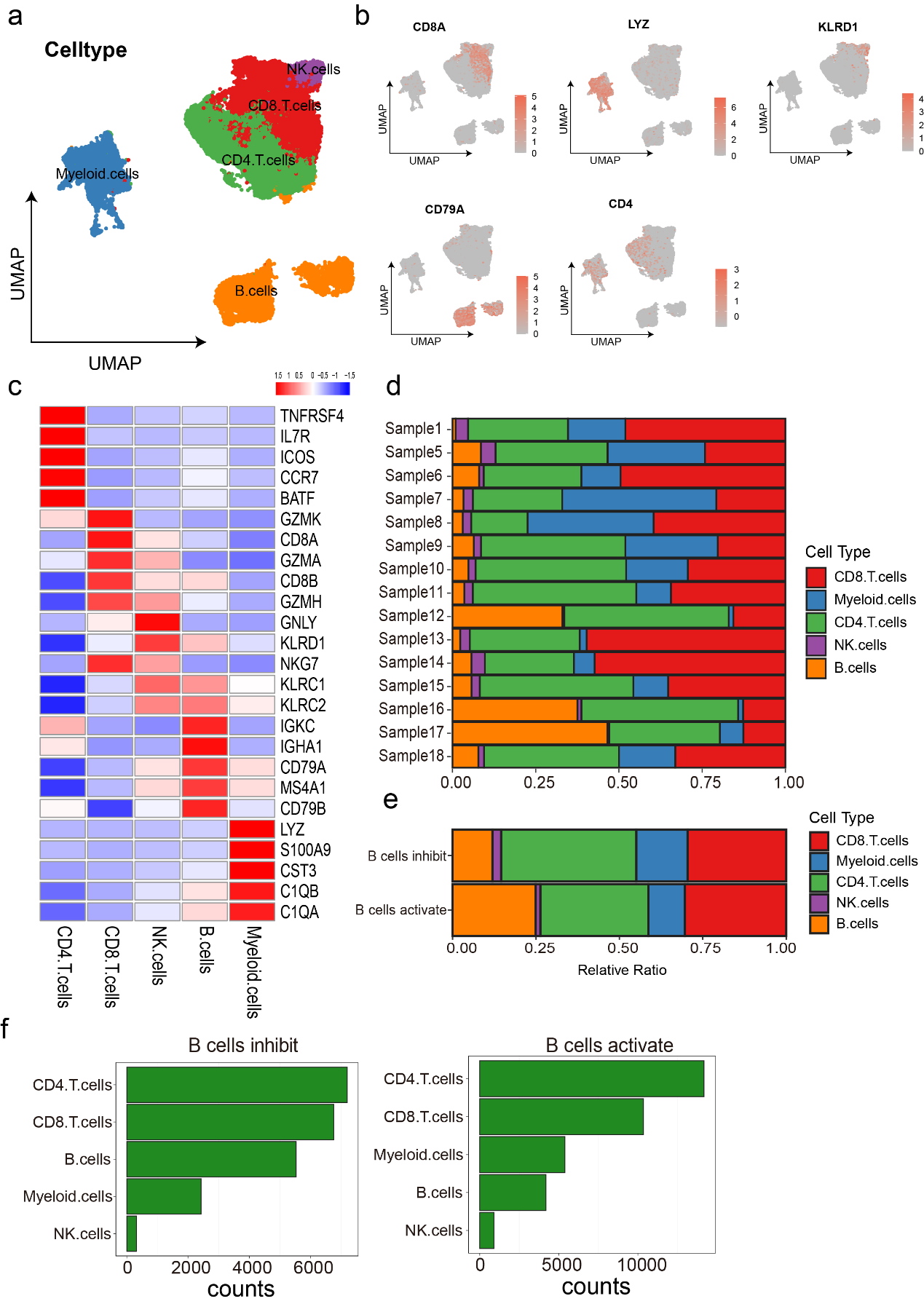


**Figure S3 Single-cell landscape of validation dataset.**

a. UMAP of tumor infiltrating immune cells (n = 60786 cells) from 15 samples in HNSCC scRNA-seq, colored by cell clusters.

b. UMAP plots showing expression of classical marker genes from immune cell clusters.

c. Heatmap of signature genes for immune cell clusters. Each cell cluster is represented by five specifically expressed genes.

d. Bar plot showing the immune cell type proportion in each HNSCC sample.

e. Bar plot showing the immune cell type proportion in B cells activation group and the B cells inhibition group.

f. Bar plots of immune cell clusters cell number in B cells activation group (left) and the B cells inhibition group (right).


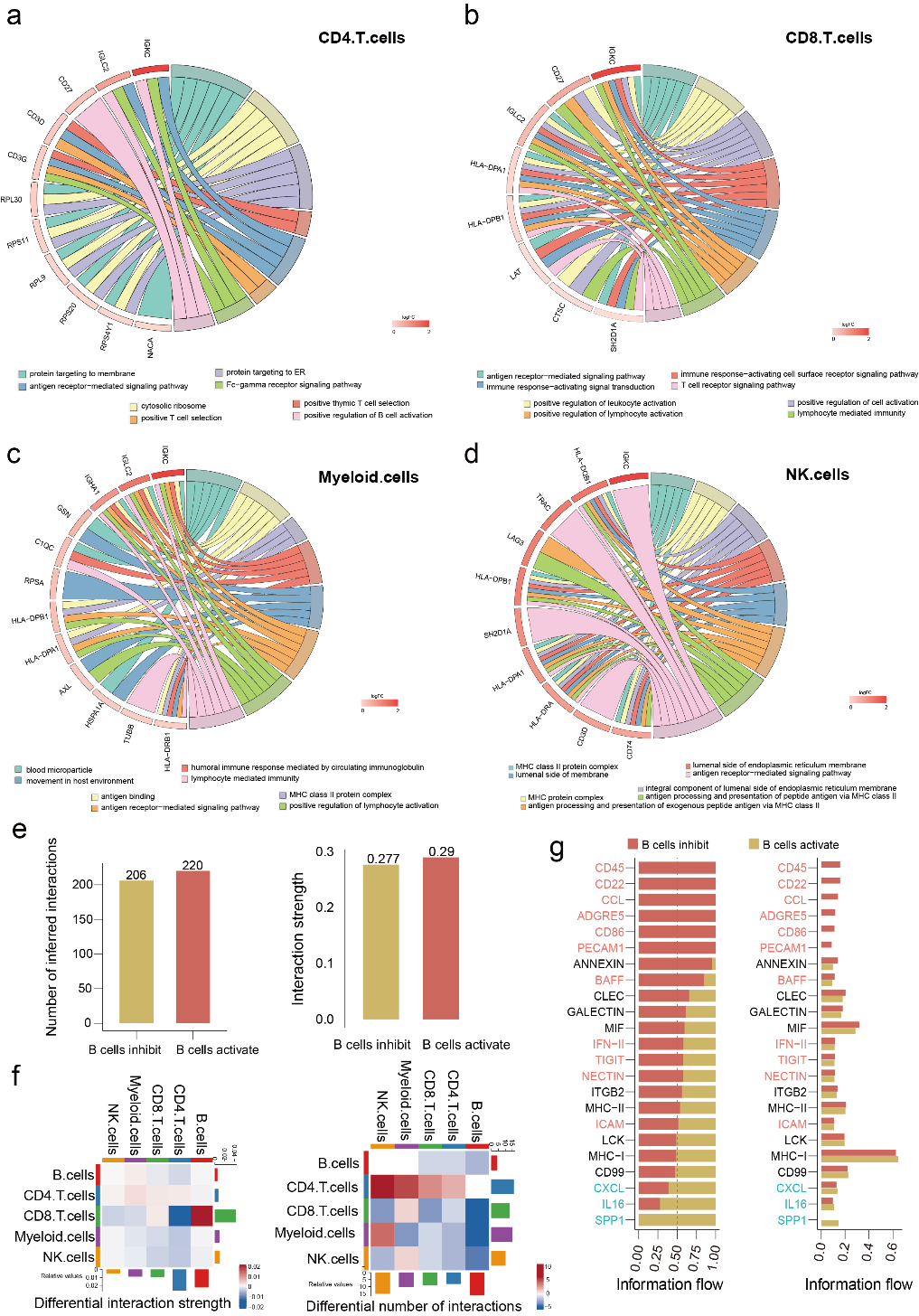


**Figure S4 GO and interaction analysis of B cells activation group in TCGA dataset.**

a-d. GO cluster plot showing a chord dendrogram of the clustering of the expression spectrum of significantly upregulated genes in CD4^+^ T cells (a), CD8^+^ T cells (b), myeloid cells (c), and NK cells (d) based on the B-cell signature genes classification.

e. The differential number of interactions (left) or interaction strength (right) in the cell-cell communication network between the two groups was shown by the circle plots.

f. Heatmap showing the differential number of interactions (left) and differential interaction strength (right) in immune clusters between the two groups. The red to dark blue gradient indicates the cell-cell communication weight values from high to low in the B cells activation group in the heatmap.

g. Bar plot showing differences in ligand-receptor interaction signaling pathways between the two groups. Signaling pathways colored in red were more enriched in B cells inhibition group, pathways colored in brown are more enriched in B cells activation group.


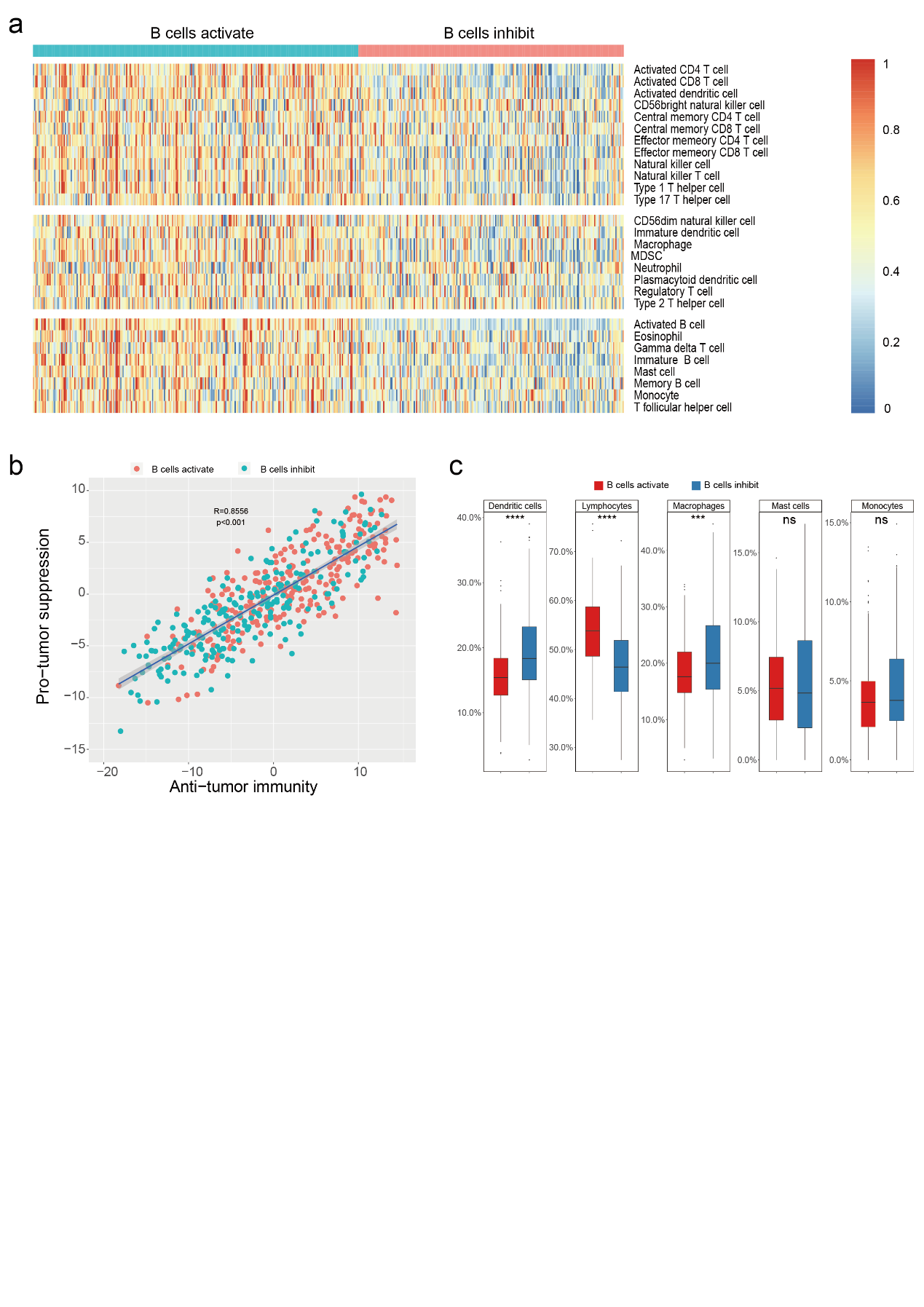


**Figure S5 Immune infiltration in the two groups of TCGA dataset.**

a. Heatmap depicts the relative infiltration of immune cell populations for patients in the B cells activation group and B cells inhibition group.

b. The correlation between pro-tumor suppression and anti- tumor immunity of TCGA HNSCC patients, colored by the sample group.

c. The comparison of 5 immune cellular infiltration components (by CIBERSORT analysis) between the two groups.
